# Correction to “A rare case of chikungunya encephalitis and its management: A case report and literature review”

**DOI:** 10.1002/ccr3.9200

**Published:** 2024-07-19

Inban P, Chandrasekaran SH, Yadav PK, Vijayakumar R, Elavia Z, Singh M. A rare case of chikungunya encephalitis and its management: A case report and literature review. *Clin Case Rep*. 2024;12:e8656. doi:10.1002/ccr3.8656

In the article entitled “A rare case of chikungunya encephalitis and its management: A case report and literature review” that was previously published in Volume 12 Issue 3 of Clinical Case Reports, the affiliation for the author—Zenia Elavia—is incorrect.

The correct affiliation is as follows:

Dr. DY Patil Medical College, Pune, India.

We apologize for this error.

